# Additional Fruit and Vegetable Vouchers for Pregnant WIC Clients: An Equity-Focused Strategy to Improve Food Security and Diet Quality

**DOI:** 10.3390/nu14112328

**Published:** 2022-06-01

**Authors:** Ronit A. Ridberg, Ronli Levi, Sanjana Marpadga, Melissa Akers, Daniel J. Tancredi, Hilary K. Seligman

**Affiliations:** 1 Center for Precision Medicine and Data Sciences, University of California Davis School of Medicine, Sacramento, CA 95817, USA; 2 Center for Vulnerable Populations, Zuckerberg San Francisco General Hospital, San Francisco, CA 94110, USA; ronli.levi@ucsf.edu (R.L.); sanjana.marpadga@ucsf.edu (S.M.); melissa.akers@ucsf.edu (M.A.); hilary.seligman@ucsf.edu (H.K.S.); 3 Division of General Internal Medicine, University of California San Francisco, San Francisco, CA 94143, USA; 4 Center for Healthcare Policy and Research, University of California Davis, Sacramento, CA 95817, USA; djtancredi@ucdavis.edu; 5 Department of Pediatrics, University of California Davis School of Medicine, Sacramento, CA 95817, USA

**Keywords:** fruit and vegetable vouchers, pregnancy, WIC, produce prescriptions, food insecurity, food purchasing behaviors

## Abstract

Women with low household income and from racial/ethnic minority groups are at elevated risk of food insecurity. Food insecurity during pregnancy is associated with overall less healthy diets, lower intake of the pregnancy-supportive nutrients iron and folate, and significant variations in diet across the course of a month. The goal of this study was to explore the impact of an ongoing $40/month supplement for fruits and vegetables (F&Vs) provided to pregnant people enrolled in the Special Supplemental Nutrition Program for Women and Children (WIC). Our primary outcome was food insecurity using the USDA 6-item survey, and our secondary outcome was dietary intake of F&Vs based on the 10-item Dietary Screener Questionnaire. Participants in intervention and comparison counties completed surveys at enrollment and approximately three months later (*n* = 609). Mean ± SD food insecurity at baseline was 3.67 ± 2.79 and 3.47 ± 2.73 in the intervention and comparison groups, respectively, and the adjusted between-group change from baseline to follow-up in food insecurity was 0.05 [95% CI: −0.35, 0.44] (*p* > 0.05). F&V intake (in cup equivalents) was 2.56 ± 0.95 and 2.51 ± 0.89 at baseline in the two groups, and the adjusted mean between-group difference in changes from baseline was −0.06 [−0.23, 0.11] (*p* > 0.05). Recruitment and data collection for this study coincided with the most intensive of America’s COVID relief efforts. Our results may indicate that small increases in highly targeted food resources make less of a difference in the context of larger, more general resources being provided to individuals and households in need.

## 1. Introduction

Pregnant people from some racial/ethnic minority groups and with low household income in the US are at a disproportionately high risk of poor birth outcomes, including preterm birth (<37 weeks gestation), compared to those who are white, not Hispanic or Asian, and to those with higher household income [1,2]. In 2019, the national preterm birth rate was 10.2%, compared with a rate of 14.4% [1] for non-Hispanic Black mothers [1]. The pre-term birth rate among non-Hispanic Black mothers is about 40% higher than the national average and more than 50% higher when compared with the pre-term birth rates of Asian (8.7%) and white (9.3%) mothers [1].

Individuals from racial/ethnic minority groups and/or with low household income are also at elevated risk of food insecurity, which is the inability for one or more household members to access enough food consistently throughout the year for an active, healthy life due to lack of resources [3]. Households with children <6 years old (where new pregnancies may be most likely to occur) are at even greater risk, with 15.3% reporting food insecurity compared with a national average of 10.5% in 2020 [3]. Food insecurity during pregnancy is related to overall less healthy diets [4], lower intake of the pregnancy-supportive nutrients iron and folate [5], and significant variations in diet across the month, with sharp reductions occurring at months’ end when food budgets have been exhausted [6]. As a result, food insecurity is likely associated with adverse birth outcomes through multiple, complex mechanisms.

The Special Supplemental Nutrition Program for Women, Infants, and Children (WIC) is designed to improve the health and wellbeing of pregnant and post-partum people, infants, and children <5 years old who are at nutritional risk by providing financial support for nutritious food purchases and nutrition education, among other services. In 2020, WIC served about 7 million Americans, including 1.6 million pregnant, breastfeeding, and post-partum people, of whom approximately 39% were Latina and 23% were Black [7]. WIC provides an available infrastructure with which to provide nutrition support to people with low incomes and at a high risk for adverse birth outcomes.

Among other foods, WIC benefits can be redeemed for fruits and vegetables. The 2009 revisions to the WIC package included an $8–11 average monthly benefit for fruits and vegetables (F&Vs) for pregnant people (as well as more whole grains and low-fat milk). An evaluation of its impact demonstrated that the package revisions were associated with reductions in maternal preeclampsia, longer gestational age, and an increased likelihood of birth weight that was appropriate for gestational age [8].

The last decade has seen an upswell of evidence that financial incentives can increase fruit and vegetable purchasing [9] as well as consumption [10,11,12] for people experiencing food insecurity and low income. In 2020, the authors published the results of a pilot study that offered pregnant WIC clients in the San Francisco Bay Area monthly vouchers of $40 for F&Vs, in addition to their existing WIC F&V benefit [13]. Among pilot participants (*n* = 592) who were food insecure at baseline, those receiving vouchers were more likely to be food secure at follow-up (23% vs. 14%, *p* = 0.04) and had larger improvements in mean daily intake frequency of total vegetables, F&Vs, salad, and non-fried potatoes (*p* < 0.05 for all categories) compared to a group of non-pregnant WIC enrollees. In addition, using a historical comparison group, odds of preterm delivery, our exploratory outcome, were 37% lower (*p* = 0.02) for those participants.

The goal of this study was to leverage the same infrastructure to address the limitations of our pilot by adding a contemporaneous comparison group of pregnant WIC clients in two neighboring counties. As in the pilot study, pregnant people enrolled in the San Francisco WIC received an ongoing supplement for F&Vs (added to standard WIC benefits). We aimed to determine the extent to which the $40 monthly vouchers reduced food insecurity and increased F&V consumption among pregnant people with low income.

## 2. Materials and Methods

### 2.1. Study Sample and Settings

Our intervention group included 304 pregnant people with low income enrolled in WIC in San Francisco, California who received $40 per month in vouchers redeemable for F&Vs in addition to their standard WIC benefit for a duration of 9 months. Our comparison group consisted of pregnant people with low income enrolled in WIC in two of San Francisco’s neighboring counties: Alameda (*n* = 226) and San Mateo (*n* = 240). These latter participants received only the standard WIC package benefits. In addition to WIC enrollment, inclusion criteria consisted of the following: currently in first or second trimester of pregnancy (<27 weeks); ability to complete electronic surveys in English, Spanish, or Chinese; intent to remain in the local area for more than 3 months; age greater than 18 years; and provision of informed consent.

### 2.2. Extenuating Circumstances

Some study plans were altered due to the onset of the COVID-19 pandemic and are reported here using adapted CONSERVE guidelines [14].

#### 2.2.1. Modifications

Enrollment was delayed for six months (from March until September 2020) to comply with university regulations governing research and to adapt the study protocol for social distancing. As WIC client services moved to being fully remote, we transitioned study implementation, WIC staff training, recruitment, enrollment, and follow-up procedures to be entirely remote. We conducted interactive webinars with WIC staff from 18 clinics within the three participating counties to train them on recruitment activities and study goals. We provided recruitment flyers, which could be disseminated via mail or digitally (e.g., email, text message, or online) to all three counties. Other adapted processes included use of an online enrollment form and a text or email-based survey dissemination strategy. In addition, fruit and vegetable vouchers were mailed to intervention county participants.

#### 2.2.2. Impacts

We initially aimed to enroll 800 total participants (50% intervention and 50% comparison); these numbers would achieve a greater than 90% power (with 2-sided alpha = 5%) to detect differences between test groups of the same magnitude and variance as those measured in our pilot study (difference-in-differences (DID) 0.88-point reduction in semicontinuous food insecurity score and DID 0.73 increase in F&V intake frequencies per day). In March 2021, federal legislation authorized states to provide a 4-month increase to the WIC Cash Value Benefit (CVB) for F&Vs up to $35/month, ending 30 September 2021, with California beginning implementation of this new policy 1 June 2021, during the study’s follow-up period. We anticipated this policy change would contaminate the study data, as our comparison groups would suddenly be eligible for a benefit that nearly matched that which was received by the intervention group. We did experience this overlap and adjusted for it in our models, but to minimize this threat, we chose to end recruitment prior to reaching our original target. Our resulting sample size achieved 80% power.

#### 2.2.3. Responsible Parties

The study team worked with WIC directors in all three counties to ensure study modifications would successfully integrate with new and rapidly changing workflows. All study procedures, including these changes as amendments, were approved by institutional review boards at both the University of California San Francisco and California State’s Committee for the Protection of Human Subjects (CPHS).

### 2.3. Intervention

Vouchers 4 Veggies, known as EatSF in San Francisco, is a financial incentive program established in 2015 that provides participants with $40 per month to purchase fresh or frozen fruits and vegetables at selected locations: a network of participating grocery stores, farmers markets, and corner stores (*n* = 30) clustered within San Francisco’s lowest-income neighborhoods. The partnership between EatSF and WIC began in 2016 and has enrolled >5500 pregnant people. EatSF enrollment occurs at WIC clinics where participants already receive prenatal services. Clients are automatically enrolled after their first pregnancy-related WIC appointment, unless they opt out of participation. EatSF provides $40 in vouchers monthly for a total of 9 months.

### 2.4. Recruitment

During participant recruitment (September 2020–June 2021), newly pregnant WIC clients (in their first or second trimester) in all three counties were informed of the study and invited to participate through flyers, emails, and/or text messages. Participants enrolled via QR code or web link. Upon confirmation of eligibility criteria via online questionnaire, they were invited to complete a baseline survey. Three months later, participants received text messages or emails (based on their stated preference during enrollment) with a link to the follow-up survey. Study follow-up occurred between December 2020 and September 2021. Though the intervention group continued to receive vouchers for an additional 6 months, we timed the follow-up survey for all participants at 3 months to minimize the proportion of participants who had already delivered their babies at the time of follow-up. Once delivery occurs, competing priorities may reduce follow-up rates and dietary intake changes substantially. However, we did allow follow-up surveys to be completed until we closed the study in September 2021. For each survey, baseline and follow-up, participants received a $20–30 gift card of their choosing from several retail options and could opt to receive the cards digitally or by post.

As part of routine, monthly quality control procedures, we cross-checked all enrolled participants with WIC agencies to confirm WIC participation (a criteria for inclusion). We identified numerous ineligible participants likely created by bots hacking the survey, which the authors describe in detail elsewhere [15]; all false survey responses (*n* = 228) were identified and removed.

### 2.5. Measures

The primary outcome of the study was food insecurity, and the secondary outcome was dietary intake of fruits and vegetables. Food insecurity was measured using the 6-item USDA food security survey module, which has 92% sensitivity and 99% specificity compared to the full 18-item module, and is validated in both English and Spanish [16]. A previously published Chinese translation, which we used in the pilot, was also used for the Chinese language survey [17,18]. We tallied affirmative responses (“often true” or “sometimes true”) for a raw score between 0 and 6, and used USDA guidelines to impute missing values [19]; we then replaced the raw score with the USDA’s Rasch-computed scale score [20] to score any affirmative responses as a continuous variable (2.86–8.48) and to better understand and account for the level of hardship experienced by households with food insecurity [21].

To assess dietary intake of F&V, we used the 10-item Dietary Screener Questionnaire (DSQ) from the National Institutes of Health, which can be converted by statistical package algorithm to cup equivalents. We chose this tool to align with metrics supported by the National Technical Assistance, Evaluation, and Information Center (NTAE), funded by the USDA Gus Schumacher Nutrition Incentive Program (GusNIP), so that we could compare our results to dozens of other nutrition incentive and produce prescription programs across the country [22]. We included response options of 1x/month, 2–3x/month, 1x/week, 2x/week, 3–4x/week, 5–6x/week, 1x/day, 2–3x/day, 4–5x/day, and 6x/day. During analysis, intake was capped at 2 times per day for all variables (except fruit juice) to align with the DSQ scoring algorithm.

### 2.6. Statistical Analysis

We used Stata Statistical Software (Version 13, College Station, TX, USA: StataCorp LP) for statistical analyses. Descriptive statistics summarized the distribution of study variables. We compared mean changes in variables of interest in the intervention group from baseline to follow-up to those of groups using a difference-in-differences (DID) approach [23] by fitting regression models, with the dependent variables being change scores (on food insecurity and on F&V intake). This method eliminated the persistent effects of stable or time non-varying characteristics and the need to control for them in the model. We did, however, include the following time-varying covariates: number of days between baseline and follow-up surveys, and whether the participant received additional food support from federal (Supplemental Nutrition Assistance Program, known as CalFresh in California, or the expanded COVID-related CVB from WIC) or municipal sources (a local grocery voucher program). We did not include time-varying characteristics for which we only had the measurement at baseline. In addition, because of discrepancies in reports of gestational age in participant surveys, we controlled instead for whether the participant was still pregnant at follow-up (yes/no).

Finally, to assess whether program effects varied by receipt of other federal food support (e.g., CalFresh or increased CVB), models were fit with and without interaction terms (each support program x treatment group) and compared using Akaike information criterion (AIC) [24]. We reported the effect sizes of the adjusted mean difference in food insecurity and F&V intake and parameter estimates (95% confidence intervals).

## 3. Results

Across intervention and comparison groups combined, participants were 55% Latina, 22% Asian or Pacific Islander, and 12% Black (Table 1). The majority of participants (56%) were between the ages of 26 and 35 years old and lived with an average of two other household members. About 62% of total participants were food insecure at baseline and 32% were enrolled in CalFresh. From the 770 participants who enrolled and completed baseline surveys, we removed from the analytic sample 161 individuals who did not complete follow-up surveys, leaving 609 participants with matched baseline and follow-up data (79% retention rate). These 609 records comprised our full analytic sample. The intervention and comparison groups were similar in many demographic characteristics. However, intervention participants were slightly older, more likely not to have completed a high school education, have no children under the age of 18 in the house, and enroll later in the study period, making them eligible for WIC’s CVB increase to $35/month. The intervention group also had a greater proportion of participants who were Asian or Pacific Islander and completed their surveys in Chinese. A greater proportion of comparison group participants took their surveys in Spanish or English and were still pregnant at follow-up. Finally, groups differed in their baseline daily consumption of fruits, vegetables, and legumes (excluding French fries), with the intervention group consuming about one-sixth of a cup equivalent more than the comparison group (0.15, *p* < 0.05).

The coefficient of the treatment effect for the adjusted mean difference in food insecurity was 0.05 (95% CI: −0.35–0.44) (Table 2). There was a reduction in food insecurity in both the treatment and comparison groups, but the two groups did not differ from each other.

We found a similar result for F&V intake. F&V intake decreased in both groups. The difference-in-differences effect size was −0.06 and non-significant (95% CI: −0.23–0.11).

Models fit with and without interaction terms did not significantly alter the results (data not shown), suggesting that program effects did not vary by receipt of CalFresh or the increased CVB alone.

## 4. Discussion

In this study, conducted during the unexpected COVID-19 pandemic, we set out to expand the findings of a promising pilot study on the use of fruit and vegetable vouchers for pregnant people; we did so by adding a contemporaneous comparison group in two neighboring counties who did not receive the vouchers. We found no significant effects from the F&V voucher on food security status and F&V intake in pregnant WIC recipients with low income. This stands in contrast to a growing body of evidence for these programs, including a 2021 systematic review and meta-analysis of 39 randomized and observational studies—providing food or resources for food—that reported statistically significant reductions in food insecurity [25], as well as a 2022 pooled analysis of 17 food pharmacy or food prescription programs which showed a significant mean increase in daily servings of F&Vs (mean = 0.77; 95% CI: 0.30 to 1.24) [26]. Perhaps most relevant to our study’s focus; the early analysis of the COVID-19-related WIC CVB augmentation demonstrates significant changes to children’s total daily F&V consumption, increasing by 1/3 cup from 2.01 cups before the CVB increase to 2.31 cups [27].

It is likely that our findings were complicated by the pandemic and subject to complex secular trends. The study period was between September 2020 and September 2021, precisely when families were struggling with the wide-reaching impacts of COVID-19 on employment, food supplies, financial stability, household food security, and regular daily routines (e.g., shopping, cooking, and food preparation). This hypothesis is consistent with a qualitative study of 182 California WIC clients during an overlapping time period that found more than a quarter reported reduced wages or work hours, job loss, and associated challenges paying for housing; nearly two-thirds reported household food insecurity; fewer were receiving school meals than prior to the pandemic (39% vs. 53%); fewer than half had received P- EBT benefits (to replace free or reduced meals that would have otherwise been consumed at school); and about a third reported challenges finding WIC-allowable foods, particularly milk, eggs, and fresh fruit [27]. For those in our intervention group, these disruptions to shopping may also have limited their ability to use their vouchers. Contributing to the limitation of voucher use as well may have been the relocation of many individuals during the pandemic. Many WIC clients were reported to be living outside of their enrollment county but continuing to receive services virtually from WIC; these clients would not be able to easily access participating retailers in San Francisco. Overall, the Vouchers 4 Veggies-Eat SF program did see lower redemption rates during this period than is typical (about 67%, compared to pre-COVID redemption rates between 74–81%), which may partially explain our null results.

Conversations with staff of our WIC partner clinics in all three counties in April 2022 also suggest that clients were in dire straits through much of the year, with a great deal of uncertainty for pregnant people about the health risks (to themselves and their pregnancy) of shopping in public spaces. Staff also reported that many families relocated and/or experienced changes to their household composition. These changes may have changed the distribution of food within a different sized household.

This period also saw much of the country’s COVID-19 relief packages made widely available, including broad financial support and expanded emergency food resources. In this complex context, our null results may demonstrate that small increases in highly targeted benefits for an intervention group (such as $40/month in F&V vouchers) make less of a difference in the midst of larger, more general resources being provided to all households in our study (e.g., multiple stimulus checks over $1000, P-EBT, SNAP benefit increases, and monthly child tax credits). This is consistent with evidence from 11 countries outside the US where cash safety nets appeared to have been more effective than food in reducing food insecurity during the pandemic [28].

This study has several limitations. In addition to the impacts of COVID-19 on individuals and households, there were also differences in how the three counties instituted COVID-19 related protocols that possibly influenced participants’ eating and shopping habits. In addition, we did not ask participants about non-food financial resources they potentially received, nor did we investigate whether there were differences in uptake of various resources by county. Second, upon data analysis we discovered 84 participants (49 in intervention group and 35 in comparison group) had enrolled in the program during their third trimester (despite multiple questions about due date and gestational age in the eligibility screener and baseline survey); we also found 213 participants to be no longer pregnant at follow-up (likely due to enrolling late in pregnancy, and/or completing their follow-up survey after 3 months). Sensitivity analyses revealed that limiting the analytic sample to participants who were still pregnant at the time of follow-up did not substantively change food security or dietary intake results (data not shown).

## 5. Conclusions

Increasing benefits for F&V purchases within the WIC program is one equity-focused strategy for supporting improved food security and diet quality among low-income pregnant people and their households. This strategy has potential for broad impact, reaching millions of pregnant people and their children each year through an already existing WIC infrastructure to reduce health disparities by targeting those most at risk. The initial 4-month increase to WIC CVB authorized in March 2021 has been extended through continuing resolution through September 2022 (with an increased benefit amount of $43/month for pregnant and postpartum individuals). It is unclear whether it will later be made permanent. While exciting evidence supporting this increase is emerging [27], more rigorous study is needed to assess the impact of these programs, particularly in the context of eligibility for other benefits. These programs deserve additional study outside of the complex policy landscape ushered in by the COVID-19 pandemic.

## Figures and Tables

**Table 1:** Participant characteristics at baseline.

Variable		Intervention (*n* = 304)	Comparison (*n* = 466)
Age, *n* (%) *			
	18–25 years old	68 (22%)	143 (31%)
	26–35 years old	177 (58%)	257 (55%)
	36–45 years old	58 (19%)	60 (13%)
	Older than 45	1 (0%)	0 (0%)
	Prefer not to answer	0 (0%)	6 (1%)
Language, *n* (%)			
	English	151 (50%)	310 (67%)
	Spanish	80 (26%)	144 (31%)
	Chinese	73 (24%)	12 (3%)
Household size, mean (SD)		3.1 (1.5)	3.2 (1.5)
Number of children under 18 in the household, mean (SD) *		1.17 (1.03)	1.42 (1.04)
	Number of children under 18 in the household, *n* (%)		
	None	95 (32%)	103 (22%)
	One	103 (34%)	151 (32%)
	Two	71 (23%)	135 (29%)
	Three	30 (10%)	69 (15%)
	Prefer not to answer	5 (2%)	8 (2%)
Household Monthly Income, *n* (%)			
	None	28 (9%)	33 (7%)
	$1–$1000	78 (26%)	119 (26%)
	$1001–$2000	89 (29%)	135 (29%)
	$2001 or more	60 (20%)	95 (20%)
	Prefer not to answer	49 (16%)	84 (18%)
Highest Educational Attainment, *n* (%) *			
	Less than or some high school	95 (31%)	104 (22%)
	High School diploma or GED	114 (38%)	218 (47%)
	Associate’s/Bachelor’s degree or trade school	74 (24%)	111 (24%)
	Advanced Degree (e.g., Master’s, Doctorate or Professional degree)	5 (2%)	10 (2%)
	Prefer not to answer	16 (5%)	23 (5%)
Receive CalFresh (Yes), *n* (%)		102 (34%)	142 (30%)
Eligible to receive additional CVB (Yes) *, *n* (%)		126 (41%)	149 (32%)
Use Emergency Food Programs (at baseline), *n* (%)			
	Never	162 (53%)	256 (55%)
	Every day or a few times per week	45 (15%)	73 (16%)
	Once a week or less	96 (32%)	136 (29%)
	Prefer not to answer	1 (0%)	1 (0%)
Race and Ethnicity, *n* (%) **			
	Latino or Hispanic	130 (44%)	278 (62%)
	Black or African American	31 (11%)	55 (12%)
	Asian or Pacific Islander	106 (36%)	58 (13%)
	White or Caucasian	15 (5%)	31 (7%)
	Native American or American Indian	0	4 (<1%)
	Other race	2 (1%)	6 (1%)
	Prefer not to answer	4 (1%)	16 (4%)
Food insecure at baseline (yes), *n* (%)		184 (62%)	282 (62%)
Days between surveys, mean (SD)		104.27 (30)	107.75 (32)
Pregnant at follow-up survey? (yes), *n* (%) *		153 (60%)	243 (68%)

* Variables differed significantly (*p* < 0.05) at baseline. ** Some totals do not equal 100% due to missing data.

**Table 2:** Mean changes in Rasch food security scores and of daily intake of fruits and vegetables (cup equivalents): intervention and comparison groups.

		Intervention		Comparison		Difference Within Groups (Follow-Up-Baseline)		Adjusted Mean Difference for the Treatment Effect ^
		Baseline	Follow-Up	Baseline	Follow-Up	Intervention	Comparison	
		Mean (SD)	Mean (SD)	Mean (SD)	Mean (SD)	Mean (SD)	Mean (SD)	Mean [95% Confidence Interval]
Fruits and vegetables, including legumes (excluding French fries) **		2.56 (0.95)	2.51 (0.89)	2.41 (1.02)	2.40 (0.91)	0.06 (0.97)	0.01 (0.94)	−0.06 [−0.23–0.11]
	Vegetables including legumes (excluding French fries) *	1.49 (0.55)	1.47 (0.46)	1.38 (0.52)	1.37 (0.44)	0.03 (0.55)	0.00 (0.48)	−0.02 [−0.11–0.06]
	Fruits	1.05 (0.56)	1.03 (0.55)	1.04 (0.65)	1.02 (0.59)	0.02 (0.67)	0.02 (0.69)	−0.01 [−0.12–0.11]
Food insecurity (Rasch score)		3.67 (2.79)	3.47 (2.73)	3.77 (2.88)	3.59 (2.84)	−0.10 (2.22)	−0.16 (2.49)	0.05 [−0.35–0.44]

* Different at baseline *p* < 0.01. ** Different at baseline *p* < 0.05. ^ covariates included in the models: the number of days between baseline and follow-up surveys; receipt of CalFresh or COVID-relief CVB including interaction terms and whether the participant was still pregnant at follow up.

## Data Availability

The data that support the findings of this study are available from the corresponding author, [R.A.R.], upon reasonable request.
